# Valorization of *Pterospartum tridentatum* (Carqueja) Stems: Influence of Extraction Methods on Phenolic Composition, Antioxidant Capacity, and Functional Bioactivity

**DOI:** 10.3390/foods15091461

**Published:** 2026-04-22

**Authors:** Tiago Barros Afonso, Teresa Bonifácio-Lopes, Eduardo M. Costa, Tiago Macedo, Joana Moreira, Juliana A. S. A. Oliveira, Manuela Pintado

**Affiliations:** 1 CBQF-Centro de Biotecnologia e Química Fina–Laboratório Associado, Escola Superior de Biotecnologia, Universidade Católica Portuguesa, Rua Diogo Botelho 1327, 4169-005 Porto, Portugal; mvlopes@ucp.pt (T.B.-L.); emcosta@ucp.pt (E.M.C.); mpintado@ucp.pt (M.P.); 2 Centre for Nanotechnology and Advanced Materials, CeNTI, Rua Fernando Mesquita 2785, 4760-034 Vila Nova de Famalicão, Portugal; tmacedo@centi.pt (T.M.); jmoreira@centi.pt (J.M.); jaoliveira@centi.pt (J.A.S.A.O.)

**Keywords:** *Pterospartum tridentatum*, *Genista tridentata*, biomass valorization, green extraction, phenolic compounds, isoflavonoids, antioxidant activity, cytotoxicity

## Abstract

*Pterospartum tridentatum* (L.) Willk. (carqueja) is widely used in traditional medicine and culinary practices in the Iberian Peninsula; however, most studies have focused on its flowers, while its stems remain largely unexplored, despite representing a significant proportion of the plant biomass. This study aimed to evaluate the potential of *P. tridentatum* stems as a source of bioactive compounds using different extraction methodologies. Aqueous, hydroethanolic, ultrasound-assisted extraction (UAE) and pressurized liquid extraction (PLE) were applied, and the resulting extracts were characterized in terms of their extraction yield, protein and carbohydrate content, phenolic composition, antioxidant capacity, antimicrobial activity, and cytotoxicity in HaCaT and Caco-2 cell lines. Phenolic profiling by LC-ESI-QqTOF-HRMS tentatively identified 37 compounds, mainly corresponding to flavonoid and isoflavonoid glycosides, with genistein derivatives representing the dominant constituents across all extracts. Although extraction yields differed among methods, phenolic profiles were broadly similar. UAE and PLE extracts showed slightly higher antioxidant activity, while antimicrobial activity was limited, with only moderate inhibition observed against *Staphylococcus epidermidis* and *Malassezia furfur*. Additionally, cytotoxicity assays indicated low toxicity. Overall, the results demonstrate that *P. tridentatum* stems represent a promising yet underutilized biomass source of phenolic compounds with antioxidant potential and low cytotoxicity under the tested in vitro conditions.

## 1. Introduction

*Pterospartum tridentatum* (L.) Willk. [=*Chamaespartium tridentatum* (L.) P. Gibbs.,] (syn. *Genista tridentata*), commonly known in Portugal as “carqueja”, is an endemic shrub of the Fabaceae family and is broadly distributed in the Iberian Peninsula and North Africa [1,2,3]. It typically reaches heights of up to 100 cm and is characterized by yellow flowers, alternate branches, and coriaceous winged stems [2,3]. *P. tridentatum* has long been used in traditional medicine, where its fresh or dried flowers are prepared as infusions, decoctions, and tonic beverages valued for their anti-inflammatory, diuretic, and depurative properties [4,5,6,7]. It is commonly used to relieve symptoms associated with colds, digestive, intestinal, and urologic problems [2,5,8,9,10]. In addition to its medicinal use, *P. tridentatum* is also used in gastronomy, for example, to flavor rice and game meat [2,11]. Previous research has reported significant antioxidant potential, as well as antimicrobial and cytotoxic activities, associated with extracts obtained from *P. tridentatum* flowers and leaves, highlighting their potential as a source of bioactive compounds for food and health-related applications [12,13,14,15]. Phytochemical studies have revealed that this species is particularly rich in phenolic compounds, especially flavonoids and isoflavones, such as genistein derivatives, which are believed to contribute to its biological activities [12,16,17].

Despite these promising findings, most previous studies have focused primarily on the flowers or aerial parts of the plant, which are the raw materials traditionally used for herbal infusions and medicinal preparations, reporting high amounts of bioactive compounds [10,11,12,14]. Additionally, toxicological studies have generally demonstrated low cytotoxicity for flower extracts, supporting the traditional consumption of carqueja preparations [3,11,14,18]. However, other plant organs, in particular stems, remain largely unexplored in terms of their chemical composition and biological potential. The investigation of stems is particularly relevant from the perspective of biomass valorization and sustainable resource use. During the harvesting and processing of *P. tridentatum*, stems are often generated as residual plant material (by-product) and have received little attention despite potentially containing valuable secondary metabolites. Exploring these underutilized plant fractions may therefore contribute to maximizing the use of available biomass while simultaneously reducing waste generation [19]. Moreover, the identification of bioactive compounds in stems could open new opportunities for the development of functional ingredients derived from this plant.

In addition to exploring new plant matrices, advances in extraction technologies have played a key role in improving the recovery of bioactive compounds from natural sources [19,20]. Conventional extraction methods, such as aqueous or hydroethanolic extraction, are widely used for the extraction of phenolic compounds. However, extraction strategies such as ultrasound-assisted extraction (UAE) and pressurized liquid extraction (PLE) have gained increasing attention as green extraction technologies. These approaches can improve extraction efficiency while reducing solvent consumption, extraction time, and energy requirements [19,20]. Despite the growing interest in these methods for the recovery of plant phenolics, their application to *P. tridentatum*, and in particular when considering stem biomass, remains poorly explored.

Therefore, the present study aimed to evaluate the potential of *P. tridentatum* stems as a source of bioactive compounds through the application of different extraction methodologies, including conventional aqueous and hydroethanolic extraction as well as assisted techniques such as UAE and PLE. The resulting extracts were characterized in terms of extraction yield, protein and carbohydrate content, phenolic composition, antioxidant capacity, antimicrobial activity, and cytotoxicity in human cell lines. By integrating phytochemical profiling with biological evaluation, this work contributes to expanding the current knowledge of *P. tridentatum* while supporting the valorization of its stem biomass as a promising source of bioactive compounds for functional and nutraceutical applications.

## 2. Materials and Methods

### 2.1. Materials

Stems of *P. tridentatum* (carqueja) were obtained as a by-product from the food sector, kindly supplied by Ervital (Castro Daire, Portugal). Upon receipt, the material was stored protected from direct light, at room temperature (below 30 °C) and at a relative humidity between 55–65%. Prior to the extraction protocols, the *P. tridentatum* stem was milled to ≤0.25 mm, using a Fritsch Pulverisette 19 cutting mill (Idar-Oberstein, Germany).

### 2.2. Extraction Methodologies

Extraction conditions are summarized in Table 1. Four extraction approaches were applied: conventional aqueous and hydroethanolic extraction, and assisted extraction using ultrasound (UAE) and pressurized liquid extraction (PLE). It should be noted that the extraction methodologies differ in several operational parameters, including solvent composition, solid-to-liquid ratio, temperature, and extraction time. Therefore, the comparison reflects the performance of each extraction system under the selected conditions rather than a fully controlled optimization of individual variables. Each extraction procedure was performed in duplicate under the specified conditions. All subsequent analytical determinations were performed in triplicate. Statistical analysis was performed as described in Section 2.11.

#### 2.2.1. Aqueous and Hydroethanolic Extraction

For the conventional solvent extractions, *P. tridentatum* stem was extracted using deionized water or ethanol–water (70:30 *v*/*v*). The aqueous extraction was carried out at a solid-to-solution ratio of 10% (*m*/*v*) for 15 min at 50 °C with agitation in a thermomixer (Vorwerk, Wuppertal, Germany). The hydroethanolic extraction was performed with a solid-to-solution ratio of 3% (*m*/*v*), with three consecutive extraction steps applied at room temperature under agitation (450 rpm). The first extraction lasted 30 min, followed by two cycles of 15 min each. After each cycle, the samples were centrifuged (Multifuge X1, Thermo Fisher Scientific, Waltham, MA, USA) and the supernatants were collected. All extracts were filtered, and the ethanol was evaporated under reduced pressure (Buchi R-210, Buchi Labortechnik AG, Flawil, Switzerland). The aqueous phase was then lyophilized to obtain a dried extract.

#### 2.2.2. Ultrasound-Assisted Extraction

Ultrasound-assisted extraction (UAE) was performed using deionized water at a solid-to-liquid ratio of 10% (*m*/*v*). The mixture was subjected to ultrasound treatment (20 kHz, programmed to work at an amplitude of 35 µm to a target energy density of 6.6 kJ/L) using an ultrasonic processor (UIP1000hdT, Hielscher, Teltow, Germany), under magnetic stirring. During ultrasound exposure, the mixture’s temperature was monitored and limited to 50 °C. Subsequently, the mixture was stirred for an additional 30 min at room temperature and then centrifuged at 9000 rpm for 10 min. The resulting supernatants were filtered through a 5 µm–13 µm paper filter and lyophilized to obtain a dried extract.

#### 2.2.3. Pressurized Liquid Extraction

Pressurized-liquid extraction (PLE) was performed using ethanol–water (50:50 *v*/*v*) as the extraction solvent at a solid-to-liquid ratio of 3% (*m*/*v*). Extraction was carried out using a 1500 mL Parr batch reactor model 5100 (Parr Instrument Company, Moline, IL, USA). The extraction vessel was purged with N_2_, followed by pressurization to 5 bar, and, subsequently, the mixture was stirred (300 rpm) for a total extraction time of 30 min at room temperature. The resulting extracts were centrifuged at 9000 rpm for 10 min, and the supernatants were filtered through a paper filter, the ethanol evaporated under pressure, and the aqueous phase lyophilized to obtain a dried extract.

#### 2.2.4. Extraction Yield

The extraction yield was calculated based on the initial amount of *P. tridentatum* stem (g) used in the extraction and the amount of dried extract (DE) obtained, as shown in the following formula:

Yield (%) = weight of DE (g)/initial weight of *P. tridentatum* stem (g) × 100

### 2.3. Total Protein Quantification

The protein content of the extracts was quantified using the micro-Kjeldahl method, according to Afonso et al. [21]. Approximately 200 mg of each extract was analyzed, and the nitrogen content was converted to protein using a conversion factor of 4.64 [22]. A blank determination was also performed. Analyses were performed in triplicate.

### 2.4. Total Carbohydrate Quantification

For the total carbohydrate content, the phenol-sulfuric acid colorimetric method was performed following the method described by [23], adapted to a 96-well microtiter plate. Briefly, each extract was reconstituted in distilled water at a final concentration of 10 mg/mL. Then, 50 µL of each sample (or its respective dilution if necessary) was mixed rapidly in a well of a 96-well microtiter plate with 150 µL of concentrated sulfuric acid (95–97%) (Honeywell, Charlotte, NC, USA). Afterwards, 30 µL of a phenol working solution (5%) was added to the mixture and heated for 5 min at 90 °C. After cooling to room temperature for an additional 5 min, the absorbance was measured at 490 nm using a Synergy H1 microplate reader (Biotek, Winooski, VT, USA) using distilled water as a blank. The results were expressed as mg glucose equivalents per g of dry extract (mg GE/g DE). Each sample was evaluated in triplicate.

### 2.5. Total Phenolic Content (TPC)

TPC was determined using the Folin–Ciocalteu method, as described by Vilas-Boas et al. [24]. Extracts were prepared in distilled water at a final concentration of 10 mg/mL. All analyses were performed in triplicate and the results expressed as mg gallic acid equivalents per g of dry extract (mg GAE/g DE). All analyses were performed in triplicate.

### 2.6. Total Flavonoid Content (TFC)

TFC was determined via the aluminum chloride colorimetric method described by Herald et al. [25]. Extracts were prepared in distilled water at a final concentration of 10 mg/mL. Catechin was used as a standard for the calibration curve, and the results were expressed as mg catechin equivalents per g of dry extract (mg CAE/g DE.) All analyses were performed in triplicate.

### 2.7. Antioxidant Activity

The antioxidant activity of *P. tridentatum* stem extracts was evaluated using the oxygen radical absorbance capacity (ORAC) assay as described in the literature with minor modifications [26]. The ORAC assay was selected as the initial functional screening due to its physiological relevance, as it measures the scavenging capacity against peroxyl radicals, which are predominant in biological systems, and considers both reaction kinetics and stoichiometry, providing a comprehensive evaluation of antioxidant activity. Lyophilized extracts were dissolved in distilled water (10 mg/mL) prior to analysis. The assay was performed in black 96-well microplates at 37 °C using fluorescein as the fluorescent probe and 2,2′-azobis(2-amidinopropane) dihydrochloride (AAPH) as the peroxyl radical generator. Fluorescence was recorded using a microplate reader (Synergy H1; excitation 485 nm, emission 528 nm). Trolox was used as the calibration standard, and the results were expressed as µmol Trolox equivalents per gram of dry extract (µmol TE/g DE). All analyses were performed in triplicate.

### 2.8. Phenolic Profiling by LC-ESI-QqTOF-HRMS

Phenolic compounds present in the carqueja extracts were analyzed using LC-ESI-QqTOF-HRMS (Bruker Daltonics, Billerica, MA, USA) following Vilas-Boas et al. [24], with some modifications. Extracts (at 10 mg/mL) were precipitated with cold methanol (−80 °C), diluted (1:20), and filtered (0.22 µm, Clarify, Phenomenex, Torrance, CA, USA). The separation was achieved in a Bruker Elute series liquid chromatograph, coupled with a UHR-QqTOF mass spectrometer with 50,000 full-sensitivity resolution (FSR) (Impact II, Bruker Daltonics, Bremen, Germany), using a BRHSC18022100 intensity Solo 2 C18 column (100 × 2.1 mm, 2.2 μm, Bruker). High-resolution mass spectrometry was used to identify the compounds. The elemental composition for the compound was confirmed according to accurate mass and isotope distribution analysis (mSigma, Bruker Daltonics). The precise mass measurement was within 5 mDa of the assigned elemental composition, and mSigma values under 20 provided confirmation. Compounds were tentatively identified by assessing the accurate mass [M-H]^−^.

### 2.9. Antimicrobial Activity

#### 2.9.1. Antibacterial Activity

The antibacterial activity of the extracts was evaluated by determining the minimum inhibitory concentration (MIC) and minimum bactericidal concentration (MBC), following the method of Afonso et al. [21]. The assay was performed against four Gram-negative bacteria (*Escherichia coli* ATCC 8739, *Pseudomonas aeruginosa* ATCC 27853, *Salmonella enterica* ATCC 13076, and *Yersinia enterocolitica* DSM 11503) and 3 Gram-positive bacteria (*Listeria monocytogenes* ATCC 15313, *Staphylococcus epidermidis* DSM 20044, and *S. aureus* ATCC 6538). Extracts were dissolved in Mueller–Hinton (MH) broth (40 mg/mL), filtered (0.22 µm), and serially diluted in microplates (20 mg/mL to 1.25 mg/mL). Bacterial suspensions (10^6^ CFU/mL) were added to each well, and plates were incubated for 24 h at 37 °C, except for *Y. enterocolitica*, which was incubated at 30 °C. Two negative controls (extract dissolved in MH broth (20 mg/mL) and MH broth only) and a positive control for each bacterium (100 μL of MH broth with 5 × 10^5^ CFU) were made. MIC values were defined as the lowest extract concentration that inhibited visible bacterial growth, while MBC values were determined by subculturing onto MH agar plates. All assays were performed in duplicate.

#### 2.9.2. Antifungal Activity

The antifungal activity of the extracts was assessed by determining the MICs and minimum fungicidal concentrations (MFCs) against four environmental filamentous fungi, namely *Aspergillus niger*, *Penicillium expansum*, *Fusarium verticillioides*, and *Cladosporium* sp., as well as the yeast *Malassezia furfur* (from the internal microbial collection of the Escola Superior de Biotecnologia da Universidade Católica Portuguesa). For the MICs’, the procedures were based on those previously described by Melo et al. [26]. The fungal concentration for the broth microdilution method was adjusted to 10^5^ spores/mL. Five serially diluted concentrations in Sabouraud Dextrose Broth (SDB) of each extract (20 mg/mL to 1.25 mg/mL) were prepared, and all were added in duplicate to a 96-well microtiter plate. A positive (100 μL of SDB with 5 × 10^4^ CFU) and a negative control (SDB only) were used. Then, the plates were incubated at 25 °C for 48 h. Afterwards, 10 μL from each well was plated onto SDA. Cultivation was conducted at 25 °C for 3 days. Regarding *M. furfur*, the determination of the MIC values followed the CLSI document M27, fourth edition [27]. MIC values were defined as the lowest extract concentration that inhibited visible fungal growth, while MFC values were determined by subculturing onto SDA plates. All assays were performed in duplicate.

### 2.10. Cell-Based Assay

#### 2.10.1. Cell Lines

Human keratinocytes (HaCaT, CLS 300493, Eppelheim, Germany) and Human Caucasian colon carcinoma epithelial cells (Caco-2, ECACC 86010202) were used in this study. HaCaT cells were cultured as monolayers at 37 °C in a humidified atmosphere containing 5% CO_2_ and 95% air. Cells were maintained in Dulbecco’s Modified Eagle Medium (DMEM, ThermoFisher Scientific, Waltham, MA, USA) containing 4.5 g/L glucose; L-glutamine without pyruvate (ThermoScientific, Waltham, MA, USA), supplemented with 10% fetal bovine serum (Lonza, Switzerland); and 1% (*v*/*v*) Penicillin–Streptomycin–Fungizone (ThermoScientific, Waltham, MA, USA). Caco-2 cells were cultured in DMEM, supplemented with 10% (*v*/*v*) fetal bovine serum, 1% (*v*/*v*) Penicillin–Streptomycin–Fungizone, and 1% non-essential amino acids. All cell lines were incubated at 37 °C in a humidified atmosphere of 5% CO_2_ and 95% air.

#### 2.10.2. Cytotoxicity Evaluation

Cytotoxicity assays were performed according to the ISO 10993-5:2009 standard, as previously described by Costa et al. [28]. Cells were grown to 80–90% confluence, detached, and seeded in a 96-well microplate at a density of 1 × 10^5^ cells/mL. After 24 h, the culture medium was replaced with fresh medium containing test samples at concentrations varying from 1000 to 62.5 µg/mL. Plain medium and medium containing 30% (*v*/*v*) DMSO were used as negative (dead) and positive (growth) controls, respectively. Following 24 h of exposure, 10 μL of PrestoBlue (ThermoFisher Scientific, USA) was added to each well, and the plates were incubated at 37 °C in the dark for 1 h. Afterwards, fluorescence (Ex: 560 nm; Em: 590 nm) was measured using a Synergy H1 microplate reader. Assays were conducted in quadruplicate, and the results were expressed as percentage inhibition of cell metabolism relative to control.

### 2.11. Statistical Analysis

The statistical analysis was performed using GraphPad Prism version 10.4.1 (GraphPad Software, La Jolla, CA, USA). The mean values and standard deviation (SD) within samples were calculated for all cases. Results were analyzed using one-way ANOVA followed by Tukey’s post hoc test, with differences considered statistically significant at *p*-values < 0.05. Additionally, multivariate analysis was performed using principal component analysis (PCA) and hierarchical clustering to evaluate the relationships between extraction methods and the measured chemical composition and bioactivity variables.

## 3. Results and Discussion

### 3.1. Yield and Chemical Composition

The results for the extraction yield, protein content, and total carbohydrate content are presented in Table 2. The highest yield was obtained with the hydroethanolic methodology (26.95%), followed by UAE, PLE, and finally aqueous extraction. This may be attributed to the ability of ethanol/water mixtures to solubilize compounds across a wider polarity range. In contrast, the lowest yield of the aqueous extract likely reflects its selectivity toward highly polar compounds. In the case of the aqueous extraction, the additional use of the ultrasound technology resulted in a higher yield when compared to the aqueous conventional extraction. Regarding the hydroethanolic extraction, the additional use of pressure did not offset the percentage of ethanol (70% to 50%) as an extraction solvent. It should be emphasized that the extraction systems evaluated in this study were not compared under fully standardized conditions. Therefore, the results reflect the performance of each method within the specific parameters applied, rather than a direct comparison of extraction technologies in isolation. These findings are consistent with previous studies on *P. tridentatum*, where higher extraction yields have been reported for alcoholic and hydroalcoholic systems compared to aqueous extraction. Ethanol-based extractions have been shown to achieve yields close to 28% to 30%, whereas aqueous extraction typically results in significantly lower values [2,8,14,17]. Extraction yields reported in the literature vary depending on the plant part, with lower efficiencies commonly observed for stems, compared with flowers [2,17]. For example, aqueous extracts of aerial parts showed reduced yields for stems, while higher values were reported for flowers during the flowering period [2].

Total protein content was relatively low in all extracts, indicating that proteins are minor constituents of carqueja stem extracts. The highest protein content was observed in the UAE (2.94 g/100 g DE), which may be explained by the ultrasound-assisted methodology, enhancing cell wall disruption and enabling the release of intracellular proteins [29]. In contrast, the lower protein content observed for the hydroethanolic and PLE extracts may be due to a reduction in protein solubility or partial precipitation of proteins in ethanol/water systems [30].

All extracts exhibited high total carbohydrate contents, confirming that carbohydrates represent a major fraction of the dry extracts. As expected, the aqueous extract showed the highest content (774.80 mg GE/g DE), consistent with the high polarity of sugars and polysaccharides. Hydroethanolic extraction resulted in the lowest values (448.18 mg GE/g DE), while the PLE and UAE methodologies resulted in intermediate carbohydrate contents.

TPC ranged from 140.80 to 166.78 mg GAE/g DE, with no significant differences observed among the extracts (Table 3). Regarding total flavonoid content, it varied between 54.25 and 67.93 mg CAE/g DE with the PLE and UAE methodologies showing higher flavonoid content, indicating that the extraction methodology may influence the relative extraction of flavonoid subclasses. Although no significant differences were observed for the TPC, the hydroethanolic extract showed a slightly higher content, which is aligned with previous studies where hydroethanolic extracts of carqueja demonstrated higher recovery of phenolics compared to infusions (42.84 vs. 34.80 mg/g dried extract) [11]. However, in another study, aqueous extracts of carqueja stems were reported to contain a high phenolic content, reaching values between 320.0 and 402.9 mg GAE/g dry matter, depending on the harvest period of the plant [2]. A methanolic extraction methodology of carqueja stems and leaves yielded a TPC of 113.60 mg GAE/g DE [10]. These differences presented in the literature values can be largely attributed to the plant part analyzed. Most previous studies evaluated flowers or whole aerial parts, whereas the present work focused specifically on stems. Plant organs are known to strongly influence phenolic accumulation, with flowers often presenting higher phenolic concentrations than vegetative structures [2]. Therefore, the lower TPC observed in the present study may reflect intrinsic compositional differences between stems and reproductive tissues rather than extraction inefficiency. The different methods employed in this study yielded similar amounts of TPC, which supports this view. However, when the extraction yield was taken into account, differences between methodologies became more evident. The hydroethanolic extraction resulted in the highest recovery efficiency (44.95 mg GAE/g plant material), indicating a greater overall extraction of phenolic compounds at the plant level, despite the relatively similar TPC values among extracts. Additionally, studies comparing wild and in vitro plant material have demonstrated significant variation in phenolic content depending on growth conditions, reinforcing that environmental factors and plant developmental stage further contribute to variability across studies [17].

Overall, the comparison between extraction methodologies in the present study suggests that while assisted techniques such as PLE and UAE may improve mass transfer, solvent polarity remains the primary determinant of phenolic recovery. Furthermore, when compared with the literature data, the plant part appears to have a stronger influence on TPC than the extraction technology itself.

Regarding the antioxidant capacity of the extracts, evaluated by the ORAC assay, the values ranged from 2665.82 to 3320.90 µmol TE/g DE, with PLE and UAE showing the highest values compared to the conventional extractions under the tested conditions. Notably, the antioxidant activity did not strictly follow TPC, as the hydroethanolic extract, despite having the highest TPC, did not exhibit the highest antioxidant value. Instead, the extracts with higher TFC (PLE and UAE) showed higher radical scavenging capacity, suggesting that flavonoids may contribute more significantly to antioxidant performance than total phenolics alone. These findings indicate that antioxidant capacity is not solely dependent on the total amount of phenolic compounds, but also on their chemical composition and the specific mechanisms involved in the assay. In particular, the ORAC assay is based on a hydrogen atom transfer mechanism, which may favor compounds such as flavonoids with high radical scavenging efficiency. In addition, potential synergistic interactions between different phenolic compounds may further enhance the overall antioxidant activity, which cannot be fully captured by TPC alone. Furthermore, flavonoids are known to exhibit strong antioxidant capacity due to their structural features, including hydroxyl groups and their ability to stabilize radical species through hydrogen atom transfer mechanisms, which are consistent with the ORAC assay. Overall, while all extracts demonstrated relevant antioxidant activity, PLE and UAE showed higher activity under the tested conditions, highlighting the importance of the extraction strategy on the antioxidant potential of the extracts.

The antioxidant activity of *P. tridentatum* extracts has been evaluated through different in vitro assays, with DPPH and TBARS among the most frequently used [6]. However, direct comparison between studies remains challenging due to the lack of methodological standardization, as differences in assay protocols, extraction solvents, and plant origin can significantly influence the reported antioxidant values [2,15,17,31].

### 3.2. Phenolic Profiling by LC-ESI-QqTOF-HRMS

The phenolic profiles of the different *P. tridentatum* stem extracts were obtained by LC-ESI-QqTOF-HRMS and are presented in Table 4. Identified peaks were grouped according to the mass (*m*/*z*) of the corresponding deprotonated molecules. Several phenolic compounds were detected, all of which were tentatively identified based on accurate mass measurements, fragmentation patterns, and comparison with the literature data. In cases where a specific tentative identification could not be assigned, compounds were classified according to their probable phenolic family or class.

Among the detected compounds, flavonoid derivatives represented the main class across all extracts, particularly flavonoid and isoflavonoid glycosides. A total of 37 peaks were tentatively identified, and their abundance varied greatly, with some compounds representing less than 1% of the total relative abundance of the extracts. The predominance of flavonoid derivatives is consistent with previous phytochemical studies describing *P. tridentatum* as a species rich in flavonoids, in which isoflavone derivatives constitute an important fraction of the phenolic composition [3,8,12,13,14,17,18,31,32]. Within this group, several genistein derivatives were detected as the main compounds in all four extracts. In particular, genistein-8-C-glucoside (*m*/*z* = 431) represented the most abundant compound, accounting for approximately 21.43–29.29% of the total phenolic abundance. Another abundant isoflavonoid was genistein-O-dihexoside (*m*/*z* = 593), accounting for approximately 10.16–17.99% of the total phenolic abundance. The other two main phenolic compounds tentatively identified in all four extracts were dihydroquercetin 6-C-hexoside (*m*/*z* = 465) and myricetin-C-hexoside (*m*/*z* = 479), accounting for approximately 9.71–14.19% and 9.24–13.90% of the total phenolic abundance, respectively. These compounds were also reported as major phenolic compounds in previous studies using aqueous, hydroethanolic, and hydromethanolic extractions, and are considered important contributors to the antioxidant properties associated with this plant [8,12,18]. In addition to these compounds, other flavonol derivatives were also detected, particularly quercetin-O-hexoside (isoquercitrin, *m*/*z* = 463), although with a relative abundance below 2%. The occurrence of other glycosylated flavonoids (*m*/*z* = 447, 461, 513, and 515) is also consistent with previous studies indicating that flavonoids in *P. tridentatum* are predominantly present as O- and C-glycosylated derivatives [8].

Most compounds were identified in all extracts; however, some exceptions were observed. The compound with *m*/*z* = 207, tentatively identified as a hydroxycinnamic acid derivative, was only detected in the PLE extract, while genistein (*m*/*z* = 269) was only identified in the hydroethanolic and PLE extracts. Additionally, quercetin (*m*/*z* = 301) was only identified in the ultrasound-assisted extract. Nonetheless, these compounds were residual, with relative abundances below 1%.

Previous phytochemical studies have mainly focused on flowers or aerial parts of *P. tridentatum*. In contrast, the present work provides, to the best of our knowledge, the first detailed phenolic profiling of *P. tridentatum* stem extracts. This distinction is particularly relevant since phenolic composition can vary significantly between plant organs due to differences in metabolic function and physiological roles. Despite these differences, stems still contain the main phenolic compounds previously reported for aerial parts of the plant, although with slight variations.

The relative abundance of phenolic compounds varied only slightly among the extraction methodologies. The PLE extract exhibited the highest total phenolic signal intensity (1.14 × 10^9^ a.u.), followed by the hydroethanolic (9.73 × 10^8^ a.u.), UAE (8.73 × 10^8^ a.u.), and aqueous (8.63 × 10^8^ a.u.) extracts. Nevertheless, the qualitative phenolic profiles of the extracts were broadly similar, with differences mainly observed in the relative abundance of individual compounds rather than in the presence or absence of major phenolic families.

Overall, the LC-ESI-QqTOF-HRMS analysis revealed that *P. tridentatum* stems contain a diverse phenolic profile dominated by isoflavonoid and flavonoid glycosides, particularly genistein derivatives. The predominance of these compounds supports the relatively high total phenolic and flavonoid contents measured in the extracts through the spectrophotometric methods described previously. Moreover, these classes of compounds are widely recognized for their antioxidant properties. In particular, genistein glycosides, as well as myricetin and dihydroquercetin derivatives, are known to exhibit strong radical scavenging activity and may contribute to the higher antioxidant capacity observed, especially for the PLE and UAE extracts.

### 3.3. Antimicrobial Activity

The antimicrobial activity of *P. tridentatum* stem extracts is presented in Table 5. Overall, the extracts exhibited limited antibacterial activity under the tested conditions. No inhibitory effects were observed against the majority of Gram-negative and Gram-positive bacteria evaluated. Only the PLE extract showed weak activity against *S. epidermidis* (MIC = 20 mg/mL), without bactericidal effect (MBC > 20 mg/mL), suggesting a predominantly bacteriostatic action at the highest concentration tested. Regarding antifungal activity, none of the extracts inhibited the growth of the filamentous fungi tested. However, some inhibitory effects were observed against *M. furfur*, with MIC values ranging from 10 to 20 mg/mL, although fungicidal concentrations were not achieved (MFC > 20 mg/mL).

These findings differ from those of previous studies reporting stronger antimicrobial activity for *P. tridentatum*. For instance, Aires et al. [13] showed dose-dependent antibacterial effects of methanolic extracts of the whole plant of *P. tridentatum* against methicillin-sensitive and methicillin-resistant *S. aureus* (MICs < 100 mg/mL), attributing this activity to their flavonoid-rich composition. Similarly, Garcia-Oliveira et al. [8] reported antibacterial and antifungal activity for hydroethanolic extracts of carqueja flowers, with minimum fungicidal concentrations as low as 0.5 to 1 mg/mL. In addition, Caleja et al. [18] also reported notable antimicrobial activity in carqueja flower infusions (MICs < 2 mg/mL) against several filamentous fungi and bacteria, reinforcing the bioactive potential of aerial parts rich in phenolic compounds. The differences between the present results and those reported in the literature may be attributed to several factors. Previous studies mainly focused on flowers or aerial parts of carqueja, whereas the present work evaluated exclusively stem extracts, which contain lower concentrations of bioactive compounds. Furthermore, solvent composition, extraction methodology, and the carbohydrate (sugar) content of the extracts may also have influenced the observed antimicrobial activity. Overall, although *P. tridentatum* has been reported to exhibit antimicrobial activity in other plant parts and extraction systems, our results indicate that stem extracts display weak antibacterial activity, with only limited antifungal effects observed against *M. furfur*. These findings reinforce the importance of plant part selection and extraction strategy in determining antimicrobial efficacy.

### 3.4. Cytotoxicity

**Figure 1:**
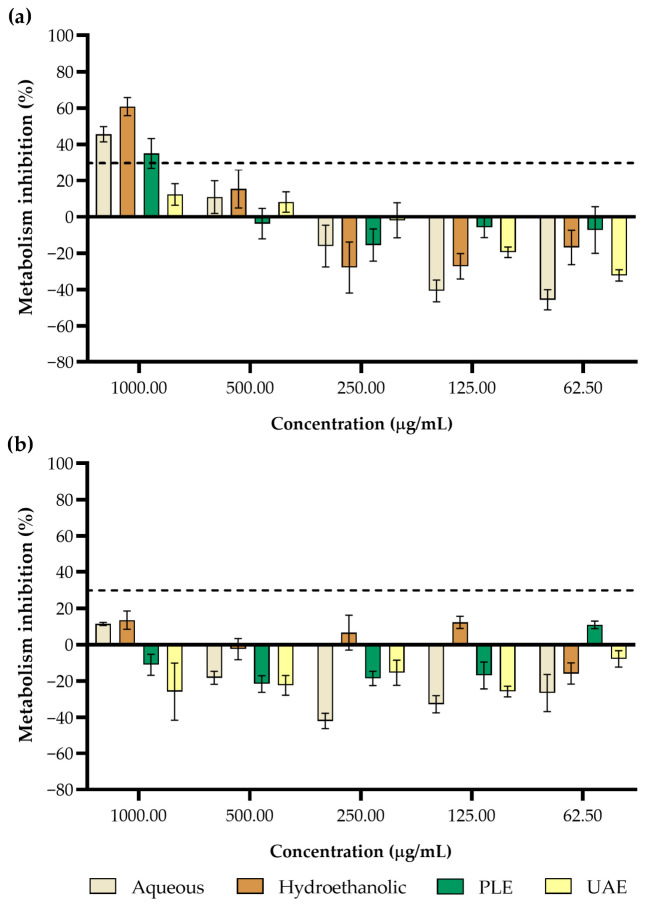
Cytotoxicity of *P. tridentatum* stem extracts at different concentrations, assessed in two cell lines: (**a**) HaCaT and (**b**) Caco-2. Dashed lines represent the 30% cytotoxicity limit as defined in the ISO 10993-5: 2009.

The cytotoxicity of the *P. tridentatum* stem extracts was evaluated in HaCaT and Caco-2 cell lines at concentrations ranging from 1000 to 62.50 mg/mL (Figure 1). Concentrations causing metabolic inhibition below 30% were considered safe according to the ISO 10993-5 standard. In the present study, most extracts did not exceed this threshold across the tested concentration range, indicating generally low cytotoxicity.

For Caco-2 cells (Figure 1b), none of the extracts induced metabolic inhibition at the tested concentrations, suggesting that the extracts did not significantly affect intestinal epithelial cell viability. Similar observations have been reported for *P. tridentatum* infusions, which showed no toxicity toward Caco-2 cells at a concentration of 2 mg/mL, while maintaining some biological activity associated with their phenolic composition [14].

Additionally, another work evaluating *P. tridentatum* flower extracts (infusion and hydroethanolic) demonstrated dose-dependent effects on Caco-2 cells, with extracts showing cytotoxicity at concentrations above 100 μg/mL and 200 μg/mL for the infusion and hydroethanolic extract, respectively [17]. However, these authors also highlighted protective effects against oxidative stress, suggesting that the extracts may interact with cellular metabolic pathways without necessarily inducing strong cytotoxicity.

In HaCaT cells (Figure 1a), however, the aqueous, hydroethanolic, and PLE extracts showed metabolic inhibition above the 30% cytotoxicity threshold at the highest tested concentration (1000 μg/mL). At concentrations below this level, all extracts remained within the non-cytotoxic range. These results indicate a concentration-dependent response, which may be related to the presence of bioactive phenolic compounds such as flavonoids and isoflavones that can influence cellular metabolism at elevated doses. No previous reports were found regarding the cytotoxicity of *P. tridentatum* extracts in this cell line. However, previous cytotoxic assessments of carqueja extracts have similarly reported low toxicity at commonly used concentrations. For example, a study evaluating flower extracts reported no cytotoxicity towards the human hepatocarcinoma cell line (HepG2) within the tested range of 125 and 375 μg/mL while also demonstrating no significant impairment of mitochondrial respiratory function or cellular viability, suggesting that the extracts can generally be regarded as safe for traditional use [3]. Additionally, analyses of plant infusions containing *P. tridentatum* reported bioactive properties such as antioxidant and antimicrobial activity without hepatotoxic effects in non-tumor cells [8,18]. In contrast to these studies, which primarily focused on flowers or aerial parts, the present work evaluates the cytotoxicity of *P. tridentatum* stem extracts, providing new information regarding the biological safety of this less-studied plant organ.

Overall, the results indicate that *P. tridentatum* stem extracts exhibit low cytotoxicity under the tested in vitro conditions toward both HaCaT and Caco-2 cell lines. Based on the ISO 10993-5 criterion (30% inhibition threshold), concentrations up to 500 µg/mL can be considered non-cytotoxic for all extracts. At 1000 µg/mL, cytotoxic effects were observed in HaCaT cells for the aqueous, hydroethanolic, and PLE extracts, while no cytotoxicity was detected for the UAE extract or in Caco-2 cells. These findings are in line with previous reports indicating that carqueja extracts can be safely used in traditional medicine, culinary applications, and for the preservation of foods against oxidative stress [3,8,14,18]. Therefore, the present results further support the potential use of *P. tridentatum* stem extracts as sources of bioactive compounds with low cytotoxicity, highlighting their potential for application in functional foods and nutraceutical formulations.

### 3.5. Principal Component Analysis

Principal component analysis (PCA) was performed to explore the relationships between extraction methods and the measured chemical and bioactivity variables, including extraction yield, protein and carbohydrate contents, TPC, TFC, antioxidant activity, and cytotoxicity parameters.

The first two principal components explained 80.76% of the total variance (PC1: 46.75%; PC2: 34.01%), indicating that the model adequately represents the dataset.

**Figure 2:**
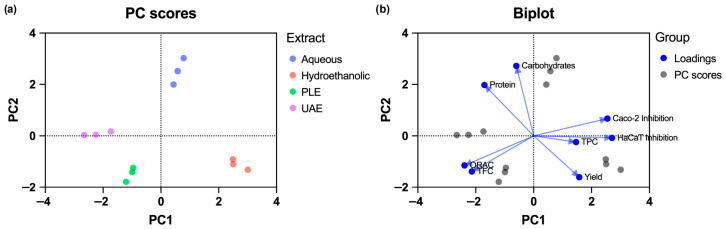
Principal component analysis (PCA) of *P. tridentatum* stem extracts based on chemical composition and bioactivity parameters. (**a**) Score plot showing the clustering of samples according to extraction method (aqueous, hydroethanolic, PLE, and UAE). (**b**) Biplot displaying the contribution of variables, including extraction yield, protein, carbohydrates, total phenolic content (TPC), total flavonoid content (TFC), antioxidant activity (ORAC), and cytotoxicity parameters (HaCaT and Caco-2 inhibition).

The PCA score plot (Figure 2a) showed a clear separation of samples according to extraction method. Hydroethanolic extracts were located on the positive side of PC1 and were associated with higher TPC, extraction yield, and cytotoxicity parameters (Figure 2b). In contrast, aqueous extracts were separated along PC2 and were mainly associated with higher carbohydrate and protein contents, reflecting the extraction of polar, non-phenolic constituents.

Extracts obtained using assisted techniques (PLE and UAE) clustered in a different region of the plot and were associated with antioxidant activity and flavonoid content (Figure 2b). This distribution suggests that, under the tested conditions, these extraction systems are associated with the recovery of compounds with higher antioxidant capacity, even when differences in TPC are relatively limited.

These results indicate that the bioactivity of *P. tridentatum* stem extracts is not solely dependent on total phenolic content but may also be influenced by the qualitative composition of the extracts and potential synergistic interactions between bioactive compounds. This observation is consistent with the fact that extracts with comparable TPC values exhibited different antioxidant responses under the tested conditions.

Overall, the multivariate analysis suggests that the extraction systems are associated with differences in antioxidant activity and phenolic composition, reinforcing the importance of selecting extraction strategies when targeting specific extract properties.

## 4. Conclusions

This study provides the first comprehensive evaluation of *Pterospartum tridentatum* stem extracts obtained using different extraction methodologies, contributing to the valorization of this underexplored plant biomass. Overall, the results demonstrated that *P. tridentatum* stems represent a relevant source of phenolic compounds, particularly flavonoid and isoflavonoid derivatives such as genistein glycosides, which were identified as the dominant constituents across all extracts.

Although the extraction methodologies differed considerably in terms of extraction yield, the qualitative phenolic profiles obtained by LC-ESI-QqTOF-HRMS were broadly similar among the four extracts. Hydroethanolic extraction resulted in the highest extraction yield and phenolic recovery, whereas PLE showed higher flavonoid content. However, the differences in total phenolic content among extraction techniques were relatively limited, indicating that more advanced extraction technologies do not necessarily lead to a substantially higher recovery of phenolic compounds in this plant matrix. Multivariate analysis demonstrated that extraction methodology influences antioxidant activity in addition to total phenolic content. Assisted extraction techniques were associated with higher antioxidant activity, whereas hydroethanolic extraction was more strongly linked to phenolic content and extraction yield. These findings suggest that antioxidant activity is not solely dependent on the total amount of phenolic compounds but may also be influenced by their qualitative composition and potential synergistic interactions. Within the broader bioactivity evaluation, antimicrobial effects were limited, whereas cytotoxicity assays indicated low cytotoxicity under the tested in vitro conditions toward both HaCaT and Caco-2 cell lines.

From a comparative perspective, hydroethanolic extraction was the most effective for maximizing extraction yield and TPC, whereas PLE and UAE were more suitable for enhancing flavonoid content and antioxidant activity under the tested conditions. In terms of cytotoxicity, all extracts exhibited low cytotoxicity, with no major differences between extraction systems. Despite these promising results, some limitations should be acknowledged. The antioxidant capacity was evaluated using a single assay (ORAC), and the application of complementary methods based on different mechanisms (e.g., electron transfer assays such as DPPH or ABTS) would provide a more comprehensive characterization. The present study should be considered exploratory in nature, providing an initial comparative evaluation of extraction systems and associated bioactivities. Future studies should therefore include additional antioxidant assays, as well as bioavailability and metabolism assessments, to better understand the biological relevance of these extracts. Furthermore, additional work focusing on the optimization of extraction conditions and the incorporation of these extracts into food or nutraceutical systems would help to support their practical application.

Overall, these findings highlight *P. tridentatum* stems as a valuable yet underutilized source of bioactive compounds with relevant antioxidant potential and low cytotoxicity under the tested in vitro conditions. From a practical perspective, the results suggest that conventional hydroethanolic extraction may be sufficient when the objective is to maximize phenolic recovery, whereas assisted extraction techniques such as PLE and UAE are more suitable when targeting enhanced antioxidant activity. This supports the valorization of carqueja stems for the development of functional foods and nutraceutical applications.

## Figures and Tables

**Table 1:** Extraction conditions for *P. tridentatum* stem extracts.

Extraction	Solvents	Solid/Liquid Ratio (% *m*/*v*)	Temperature (°C)	Time (min)	Agitation/Energy Input	Pressure
Aqueous	H_2_O	10%	50 °C	15	Thermomixer (agitation)	Atmospheric
Hydroethanolic	EtOH:H_2_O (70:30 *v*/*v*)	3%	RT	30 + 15 + 15	450 rpm	Atmospheric
UAE	H_2_O	10%	RT	Ultrasound + 30 stirring	20 kHz, 35 µm, 6.6 kJ/L	Atmospheric
PLE	EtOH:H_2_O (50:50 *v*/*v*)	3%	RT	30	300 rpm	5 bar

**Table 2:** Extraction yield, protein, and total carbohydrate content of *P. tridentatum* stem extracts. Results are presented as mean ± SD. Different letters in each column stand for statistical differences (*p*-value < 0.05).

Extract	Yield (%)	Protein (g/100 g DE)	Carbohydrates (mg GE/g DE)
Aqueous	8.37 ± 0.17 ^a^	2.58 ± 0.06 ^a^	774.80 ± 43.04 ^a^
Hydroethanolic	26.95 ± 0.54 ^b^	1.32 ± 0.03 ^b^	448.18 ± 17.26 ^b^
PLE	16.10 ± 0.14 ^c^	1.43 ± 0.11 ^b^	513.88 ± 13.72 ^c^
UAE	14.72 ± 0.09 ^c^	2.94 ± 0.04 ^c^	595.03 ± 23.28 ^d^

**Table 3:** Results for total phenolic content, recovery efficiency, total flavonoid content, and antioxidant activity (ORAC) of *P. tridentatum* stem extracts. Recovery efficiency was calculated by combining extraction yield and TPC values and expressed as mg GAE per g of plant material. Results are presented as mean ± SD. Different letters in each column stand for statistical differences (*p*-value < 0.05).

Extract	TPC (mg GAE/g DE)	Recovery Efficiency (mg GAE/g Plant Material)	TFC (mg CAE/g DE)	ORAC (µmol TE/g DE)
Aqueous	150.14 ± 12.04 ^a^	12.57 ± 1.01 ^a^	54.25 ± 2.76 ^a^	2665.82 ± 127.18 ^a^
Hydroethanolic	166.78 ± 27.78 ^a^	44.95 ± 7.28 ^b^	56.80 ± 1.98 ^a^	2672.77 ± 100.26 ^a^
PLE	140.80 ± 10.26 ^a^	22.67 ± 1.65 ^c^	67.93 ± 2.78 ^b^	3320.90 ± 45.62 ^b^
UAE	147.29 ± 20.69 ^a^	21.68 ± 3.05 ^c^	64.70 ± 0.92 ^b^	3147.13 ± 15.75 ^b^

**Table 4:** Phenolic compounds tentatively identified in the different *P. tridentatum* extracts by LC-ESI-QqTOF-HRMS.

Peak	RT (min)	*m*/*z* Calc. [M-H]^−^	MS2 [M-H]^−^	Proposed Formula	Tentative Identification	Extracts; Peak Area–a.u. (Relative Abundance-%)			
						Aqueous	Hydroethanolic	PLE	UAE
1	12.7	465	125, 167 *, 197, 317, 345, 375	C_21_H_22_O_12_	Dihydroquercetin 6-C-hexoside	1.22 × 10^8^ (14.19)	9.45 × 10^7^ (9.71)	1.15 × 10^8^ (10.06)	1.12 × 10^8^ (12.87)
2	14.3	579	89, 101, 113, **119**, 161, 179, 209	C_24_H_36_O_16_	Phenolic glycoside	n.d.	n.d.	n.d.	8.58 × 10^6^ (0.98)
3	15.4	417	176, 194, **209**	C_18_H_26_O_11_	Phenolic glycoside	2.74 × 10^7^ (3.18)	2.01 × 10^7^ (2.06)	2.96 × 10^7^ (2.60)	2.76 × 10^7^ (3.16)
4	17.3	623	**415**	C_28_H_32_O_16_	Flavonol glycoside	n.d.	1.37 × 10^7^ (1.40)	1.48 × 10^7^ (1.30)	n.d.
5	17.6	653	**445**	C_29_H_34_O_17_	Flavonoid glycoside	n.d.	n.d.	8.14 × 10^6^ (0.72)	n.d.
6	18.6	593	310, **473**	C_27_H_30_O_15_	Genistein-O-dihexoside	1.55 × 10^8^ (17.99)	1.64 × 10^8^ (16.84)	1.73 × 10^8^ (15.21)	8.87 × 10^7^ (10.16)
7	20.0	479	167, **359**	C_22_H_24_O_12_	Myricetin-C-hexoside	1.14 × 10^8^ (13.17)	8.99 × 10^7^ (9.24)	1.17 × 10^8^ (10.27)	1.21 × 10^8^ (13.90)
8	20.7	207	**96**, 105, 121, 149, 177	C_11_H_12_O_4_	Hydroxycinnamic acid derivative	n.d.	n.d.	8.14 × 10^6^ (0.71)	n.d.
9	21.1	639	**431**	C_28_H_32_O_17_	Flavonoid glycoside	2.83 × 10^7^ (3.28)	9.11 × 10^7^ (9.36)	8.75 × 10^7^ (7.68)	n.d.
10	22.8	447	299, **327**	C_21_H_20_O_11_	Flavonoid C-glycoside	7.54 × 10^6^ (0.87)	7.91 × 10^6^ (0.81)	8.58 × 10^6^ (0.75)	1.27 × 10^7^ (1.46)
11	24.3	479	167, **359**	C_22_H_24_O_12_	Myricetin-C-hexoside	7.20 × 10^6^ (0.83)	n.d.	8.00 × 10^6^ (0.70)	8.20 × 10^6^ (0.94)
12	24.4	415	89, **99**, 101, 113, 119, 131, 161, 191	C_19_H_28_O_10_	Phenolic glycoside	7.31 × 10^6^ (0.85)	n.d.	n.d.	n.d.
13	25.2	433	89, 101, 113, **119**	C_19_H_14_O_12_	Ellagic acid pentoside	4.10 × 10^7^ (4.75)	2.77 × 10^7^ (2.85)	4.48 × 10^7^ (3.93)	4.72 × 10^7^ (5.42)
14	28.0	431	283, **311**	C_21_H_20_O_10_	Genistein-8-C-glucoside	2.04 × 10^8^ (23.59)	2.21 × 10^8^ (22.73)	2.44 × 10^8^ (21.43)	2.56 × 10^8^ (29.29)
15	28.5	461	298, 326, **341**	C_22_H_22_O_11_	5,5′-Dihydroxy-3′-methoxy-isoflavone-7-O-β-glucoside	2.85 × 10^7^ (3.30)	4.14 × 10^7^ (4.29)	4.49 × 10^7^ (3.95)	3.56 × 10^7^ (4.08)
16	29.1	431	**268**, 311	C_21_H_20_O_10_	Genistein-8-C-glucoside	n.d.	7.02 × 10^6^ (0.72)	8.42 × 10^6^ (0.74)	n.d.
17	29.4	415	**252**	C_21_H_20_O_9_	Phenolic glycoside	1.05 × 10^7^ (1.22)	n.d.	6.83 × 10^6^ (0.60)	1.01 × 10^7^ (1.16)
18	30.3	461	298, 326, **341**	C_22_H_22_O_11_	5,5′-Dihydroxy-3′-methoxy-isoflavone 7-O-β-glucoside	n.d.	n.d.	4.51 × 10^6^ (0.40)	n.d.
19	30.7	463	**300**	C_21_H_20_O_12_	Quercetin-O-hexoside (isoquercitrin)	6.72 × 10^6^ (0.78)	1.75 × 10^7^ (1.80)	2.16 × 10^7^ (1.90)	1.00 × 10^7^ (1.15)
21	30.9	431	**268**	C_21_H_20_O_10_	Genistein 7-O-glucoside (genistin)	n.d.	1.55 × 10^7^ (1.60)	1.44 × 10^7^ (1.26)	n.d.
22	31.1	463	300, 343	C_21_H_20_O_12_	Quercetin-O-hexoside (isoquercitrin)	n.d.	n.d.	1.47 × 10^7^ (1.29)	n.d.
23	32.5	431	**268**	C_21_H_20_O_10_	Genistein 7-O-glucoside (genistin)	7.94 × 10^7^ (9.20)	4.80 × 10^7^ (4.93)	5.71 × 10^7^ (5.02)	5.53 × 10^7^ (6.34)
24	33.2	431	**268**, 311	C_21_H_20_O_10_	Genistein-8-C-glucoside	n.d.	4.57 × 10^6^ (0.47)	5.20 × 10^6^ (0.46)	n.d.
25	33.3	287	107, **125**, 151, 177	C_15_H_12_O_6_	Flavanone	n.d.	3.83 × 10^6^ (0.39)	n.d.	7.19 × 10^6^ (0.82)
26	33.9	513	**393**, 423	C_26_H_26_O_11_	Flavonoid C-glycoside	1.12 × 10^7^ (1.30)	1.73 × 10^7^ (1.77)	2.43 × 10^7^ (2.13)	1.61 × 10^7^ (1.84)
27	34.6	269	107, 135, 159, 183, 201	C_15_H_10_O_5_	Genistein	n.d.	4.31 × 10^6^ (0.44)	4.93 × 10^6^ (0.43)	n.d.
28	34.8	283	184, 196, **240**, 268	C_16_H_12_O_5_	4‘-O-Methylgenistein (biochanin A)	5.05 × 10^6^ (0.59)	1.16 × 10^7^ (1.19)	1.33 × 10^7^ (1.17)	1.53 × 10^7^ (1.75)
29	35.3	445	282, 297, **325**	C_22_H_22_O_10_	Swertisin	n.d.	4.19 × 10^6^ (0.43)	5.67 × 10^6^ (0.50)	3.57 × 10^6^ (0.41)
30	35.6	515	282, **353**, 445	C_26_H_28_O_11_	Prenylated isoflavone glucoside	n.d.	4.81 × 10^6^ (0.49)	5.96 × 10^6^ (0.52)	n.d.
32	36.0	253	91, 133, 209, 224	C_15_H_10_O_4_	Daidzein	7.93 × 10^6^ (0.92)	1.24 × 10^7^ (1.28)	1.30 × 10^7^ (1.14)	2.22 × 10^7^ (2.54)
33	36.5	285	133, 149, **175**, 199, 217, 241, 268	C_15_H_10_O_6_	Kaempferol	n.d.	1.09 × 10^7^ (1.12)	1.30 × 10^7^ (1.14)	4.69 × 10^6^ (0.54)
34	36.8	285	135, 149, 185, 229, 257	C_15_H_10_O_6_	Scutellarein	n.d.	1.45 × 10^7^ (1.49)	1.07 × 10^7^ (0.94)	5.71 × 10^6^ (0.65)
35	36.9	369	**293**, 351	C_20_H_18_O_7_	Prenylated flavonoid	n.d.	6.27 × 10^6^ (0.64)	n.d.	n.d.
36	37.7	301	121, **151**, 183, 245	C_15_H_10_O_7_	Quercetin	n.d.	n.d.	n.d.	4.69 × 10^6^ (0.54)
37	37.9	369	**293**, 351	C_20_H_18_O_7_	Prenylated flavonoid	n.d.	1.87 × 10^7^ (1.92)	1.19 × 10^7^ (1.05)	n.d.
					**Total Phenolic Area (a.u.)**	8.63 × 10^8^	9.73 × 10^8^	1.14 × 10^9^	8.73 × 10^8^

* **Bold** represents the main fragment; [M-H]^−^ is the deprotonated molecular ion; n.d.—not detected.

**Table 5:** Antimicrobial activity of the different carqueja stem extracts.

Microorganisms Bacteria	MIC and MBC (mg/mL)							
	Aqueous		Hydroethanolic		PLE		UAE	
	MIC	MBC	MIC	MBC	MIC	MBC	MIC	MBC
*E. coli*	n.d. *	n.d.	n.d.	n.d.	n.d.	n.d.	n.d.	n.d.
*P. aeruginosa*	n.d.	n.d.	n.d.	n.d.	n.d.	n.d.	n.d.	n.d.
*S. enterica*	n.d.	n.d.	n.d.	n.d.	n.d.	n.d.	n.d.	n.d.
*Y. enterocolitica*	n.d.	n.d.	n.d.	n.d.	n.d.	n.d.	n.d.	n.d.
*S. aureus*	n.d.	n.d.	n.d.	n.d.	n.d.	n.d.	n.d.	n.d.
*S. epidermidis*	n.d.	n.d.	n.d.	n.d.	20	>20	n.d.	n.d.
*L. monocytogenes*	n.d.	n.d.	n.d.	n.d.	n.d.	n.d.	n.d.	n.d.
**Fungi**	MIC	MFC	MIC	MFC	MIC	MFC	MIC	MFC
*A. niger*	n.d.	n.d.	n.d.	n.d.	n.d.	n.d.	n.d.	n.d.
*Cladosporium* sp.	n.d.	n.d.	n.d.	n.d.	n.d.	n.d.	n.d.	n.d.
*F. verticillioides*	n.d.	n.d.	n.d.	n.d.	n.d.	n.d.	n.d.	n.d.
*P. expansum*	n.d.	n.d.	n.d.	n.d.	n.d.	n.d.	n.d.	n.d.
*M. furfur*	20	>20	10	>20	10	>20	20	>20

* n.d.—no inhibition detected at the highest tested concentration of 20 mg/mL.

## Data Availability

The original contributions presented in this study are included in the article. Further inquiries can be directed to the corresponding author.

## Abbreviations

The following abbreviations are used in this manuscript:

AAPH	2,2′-Azobis(2-amidinopropane) dihydrochloride
ANOVA	Analysis of variance
ATCC	American Type Culture Collection
CAE	Catechin equivalents
CFU	Colony-forming units
CLSI	Clinical and Laboratory Standards Institute
DE	Dry extract
DMEM	Dulbecco’s Modified Eagle Medium
DMSO	Dimethyl sulfoxide
EtOH	Ethanol
GE	Glucose equivalents
GAE	Gallic acid equivalents
LC-ESI-QqTOF-HRMS	Liquid chromatography–electrospray ionization–quadrupole time-of-flight high-resolution mass spectrometry
MBC	Minimum bactericidal concentration
MeOH	Methanol
MFC	Minimum fungicidal concentration
MH	Mueller–Hinton
MIC	Minimum inhibitory concentration
*m*/*z*	Mass-to-charge ratio
ORAC	Oxygen radical absorbance capacity
PLE	Pressurized liquid extraction
RT	Retention time
SDA	Sabouraud dextrose agar
TFC	Total flavonoid content
TPC	Total phenolic content
TE	Trolox equivalents
UAE	Ultrasound-assisted extraction
